# *R. vesicarius* L. exerts nephroprotective effect against cisplatin-induced oxidative stress

**DOI:** 10.1186/s12906-021-03398-9

**Published:** 2021-09-04

**Authors:** Md. Mahmudul Hasan, Most. Sayla Tasmin, Ahmed M. El-Shehawi, Mona M. Elseehy, Md. Abu Reza, Ariful Haque

**Affiliations:** 1 Molecular Biology and Protein Science Laboratory, Department of Genetic Engineering and Biotechnology, Faculty of Life and Earth Sciences, University of Rajshahi, Rajshahi, 6205 Bangladesh; 2 Molecular Pathology Laboratory, Institute of Biological Sciences, University of Rajshahi, Rajshahi, 6205 Bangladesh; 3 Department of Biotechnology, College of Science, Taif University, P.O. Box 11099, Taif, 21944 Saudi Arabia; 4 Department of Genetics, Faculty of Agriculture, Alexandria University, Alexandria, 21545 Egypt

**Keywords:** Cisplatin, *R. vesicarius*, Mice, Kidney, HK-2 cells, Oxidative stress, NQO1 gene

## Abstract

**Background:**

Cisplatin is an outstanding anticancer drug, but its use has been decreased remarkably due to sever nephrotoxicity. *R. vesicarius* L. is a leafy vegetable that is evident with anti-angeogenic, anti-inflammatory, anti-proliferative, hepatoprotective, and nephroprotective potential. Therefore, this study was designed to inspect its methanol extract (RVE) for possible nephroprotective effect.

**Methods:**

Primarily, in vitro antioxidant activity of RVE was confirmed based on 2, 2-diphenyl-1-picrylhydrazyl (DPPH) free radical scavenging aptitude. Thereafter, Swiss Albino male mice were treated with cisplatin (2.5 mg/kg) for 5 successive days to induce nephrotoxicity. Recovery from nephrotoxicity was scrutinized by treating the animals with RVE (25, 50, and 100 mg/kg) intraperitoneally (*i.p.*) for the next 5 consecutive days. After completion of treatment, mice were sacrificed and kidneys were collected. Part of it was homogenized in sodium phosphate buffer for evaluating malondialdehyde (MDA) level, another part was used to evaluate gene (NQO1, p53, and Bcl-2) expression. Moreover, the hydrogen peroxide (H_2_O_2_) neutralizing capacity of RVE was evaluated in HK-2 cells in vitro. Finally, bioactive phytochemicals in RVE were determined using gas chromatography–mass spectrometry (GC-MS).

**Results:**

RVE showed in vitro antioxidant activity in a dose-dependent fashion with 37.39 ± 1.89 μg/mL IC_50_ value. Treatment with RVE remarkably (*p* < 0.05) decreased MDA content in kidney tissue. Besides, the expression of NQO, p53, and Bcl-2 genes was significantly (*p* < 0.05) mitigated in a dose-dependent manner due to the administration of RVE. RVE significantly (*p* < 0.05) reversed the H_2_O_2_ level in HK-2 cells to almost normal. From GC-MS, ten compounds including three known antioxidants “4H-Pyran-4-one, 2, 3-dihydro-3,5-dihydroxy-6-methyl-”, “Hexadecanoic acid”, and “Squalene” were detected. The extract was rich with an alkaloid “13-Docosenamide”.

**Conclusion:**

Overall, RVE possesses a protective effect against cisplatin-induced kidney damage.

## Introduction

Oxidative stress is the result of disproportion between the formation of reactive oxygen species (ROS) and regular antioxidant defense mechanisms [1]. Regular biochemical reactions, frequent exposure to the unfavorable environment, and elevated intake of xenobiotics result in ROS production [1]. ROS interact with the cysteine residues of redox-sensitive signaling molecules including transcription factors, protein tyrosine phosphatases, and protein kinases; consequently, oxidation of thiol groups on these residues guide to alterations of the targeted proteins, biological actions, signaling capacities, immunity, and supplementary cell live/dead paradigms [2]. Oxygen-containing chemical species having reactive properties are known as ROS which includes free radicals and non-radical molecules such as superoxide and H_2_O_2_, respectively [3]. Oxidative stress induced by ROS is linked with the etiology of numerous diseases including cancer. Acute myeloid leukemia (AML) is a cancerous growth of blood cells within the bone marrow. The cellular and molecular events underlying AML include DNA damage, clonal propagation, increased cell death, and further genetic instability, which are the result of ROS-induced oxidative stress [4]. Human physiology has been gifted with numerous mechanisms that can generate antioxidants to exert protection against oxidative stress leading to protect cells from toxic effects and serve to disease prevention [5]. However, cells develop endogenous mechanisms to counteract oxidative stress and conserve required ROS [6].

NAD(P)H:quinone oxidoreductase 1 (NQO1) is a flavoenzyme [7] that can catalyze two or four-electron reduction and utilizes this property to detoxify quinines [8]. It can protect cells from oxidative damage by keeping redox cycling aside and by reducing the production of free radicals [8]. Beside xenobiotic detoxification, NQO1 is also involved in superoxide neutralization, modulation of p53 proteasomal degradation [9], Bcl-2 inhibition [10], and enhance susceptibility to cell injury [11].

Cisplatin is the first Food and Drug Administration (FDA)-approved platinum-based anticancer drug [12]. Cisplatin exerts apoptosis by inducing oxidative stress and overexpression of tumor suppressor gene p53 [12]. Several adverse effects including nephrotoxicity, hepatotoxicity, gastrotoxicity, ototoxicity, myelosuppression, and neurotoxicity are the result of cisplatin-induced oxidative stress [12]. These side effects have remarkably decreased the use of cisplatin though it has outstanding anticancer activity. Cisplatin is well known to induce oxidative stress and suppress NQO1 gene in mice kidneys [13]. Therefore, searching and validation for effective natural sources of antioxidants are becoming an area of awareness. Intake of plant-derived dietary antioxidants such as flavonoids, carotenoids, and phenolic compounds may lead to protection against cardiovascular diseases, cataracts, and cancer [14].

*R. vesicarius* (*Polygonaceae*) is known as “Takpalong/Chukapalong/Amlabetom” in Bengali [15]. It grows in the desert and semi-desert areas of Asia, Australia, and North Africa [16]. It is a little-studied endangered plant in Bangladesh. In Bangladesh, people consume the whole plant as a vegetable after cooking with salt, different spices, and oil. Sometimes, people use only the fresh leaves in mixed salad as an alternative to lettuce. The raw leaf is slightly sour, but it becomes highly sour after cooking. Moreover, a little number of leaves are usually being mixed in fish dishes during cooking to have a mildly acidic taste.

This plant is being used as a vegetable and medicinal herb worldwide [17]. The leaves and seeds are used as an antidote for snake and scorpion venom, respectively [17]. In folk treatment, *R. vesicarius* has long been used in treating hepatic diseases, bad digestion, constipation, piles, vomiting, flatulence, heart troubles, pains, spleen disorders, dyspepsia, toothache, bronchitis, asthma, scabies, leucoderma, and as laxative, stomachic, appetizer, tonic, diuretic, and analgesic [18]. This plant comprises numerous biologically important compounds including flavonoids, anthraquinones, carotenoids, vitamins, lipids, and organic acids, which are well known as antioxidant, antimicrobial, and anticancer agents [19]. Every part of this plant contains quercetin (flavonoids) in an elevated amount [15]. This plant contains 0.25 mg vitamin A, 1.33 mg vitamin C, 2.37 mg vitamin E [15], 3.38 mg flavonoids, and 5.66 mg polyphenols [20] per 100 g dry weight.

Shahat and colleagues [21] showed anti-angiogenic and anti-proliferative effects of methanol (80%) extract of *R. vesicarius* aerial part against hepatocellular carcinoma in rat model. Another study showed in vitro anti-angiogenic potential of *R. vesicarius* extract [22]. Methanol extract of whole *R. vesicarius* exerts protection against carbon tetrachloride-induced hepatotoxicity in vivo [23]. Anti-inflammatory effect in rabbit has been evident by methanolic leaf extract of *R. vesicarius* [24]*.* A recent study [25] reported in vivo nephroprotective effect of fractionated ethanolic *R. vesicarius* extract against gentamicin and potassium dichromate toxicity.

Keeping the above information in consideration, we aimed to inspect the effect of *R. vesicarius* extracts (RVE) in terms of recovery from cisplatin-induced nephrotoxicity through maintaining NQO1 gene expression in animal model.

## Materials and methods

### Chemicals and reagents

Cisplatin and 2, 2-diphenyl-1-picrylhydrazyl (DPPH) were purchased from SIGMA-ALDRICH (USA). Creatinine Colorimetric Assay Kit (product ID – 700,460) was purchased from Cayman Chemical (USA). Dulbecco’s Modified Eagle’s Medium (DMEM), fetal bovine serum (FBS), and antibiotic (10,000 U/mL penicillin and 10,000 μg/mL streptomycin) were purchased from Gibco (Gibco Laboratories, USA). ROS-Glo™ H_2_O_2_ Assay kit and GoTaq® qPCR Master Mix were obtained from Promega (USA). Reverse-transcription kit TIANScript M-MLV was purchased from TIANGEN (China) and primers from IDT (Integrated DNA Technologies, Malaysia). All other chemicals and reagents used in this experiment were of analytical grade.

### Plant sample collection and extract preparation

Fresh *R. vesicarius* plants were purchased from a local market at Sonadighi, Rajshahi, Bangladesh. Plant specimen was identified and authenticated by Dr. Ahmad Humayan Kabir, Department of Botany, University of Rajshahi, Bangladesh. A specimen under voucher no. 00095 was stored in the herbarium of the Department of Botany, University of Rajshahi. The aerial parts of the plant were cleaned, dried at 37 °C, ground to coarse powder using an electronic dryer, and stored in a sealed container at 4 °C. The fine powder (10 g) was dissolved in methanol (500 mL). The content was sonicated (Soniprep 150, China) at 20 kHz for 10 min. Filtration of the extract was carried out by using Glass Fiber Filter paper (Macherey NAGEL, GmBH, German) with DURAN® Filtering Apparatus (German). Finally, the filtrate was concentrated using a freeze dryer (VirTis BenchTop Pro, SP SCIENTIFIC, USA). The extract was finally named RVE.

### In-vitro antioxidant activity test

In-vitro antioxidant capacity of RVE was carried out based on scavenging of DPPH as described previously [26] with little modification. DPPH radical scavenging ability of RVE was assessed based on converting the purple colour of DPPH to yellow colour. The reaction mixture in each micro-centrifuge tube (2 mL) consisted 950 μL methanolic solution of DPPH radicals (0.1 mM) and 50 μL RVE from five different concentration (200, 500, 1000, 2000, and 4000 μg/mL methanol) to make final concentrations of 10, 25, 50, 100 and 200 μg/mL. Another tube containing 50 μL methanol and 950 μL methanolic solution of DPPH was kept as control. The test tubes were left for 30 min in dark place. The absorbance of the mixtures was taken at 517 nm using GENESYS 10S UV-VIS spectrophotometer (Thermo SCIENTIFIC, USA). Finally, the percentage of radical scavenging activity (RSA) was calculated based on discoloration of DPPH using the following formula-.

%RSA = [(A_DPPH_ − A_RVE_)/A_DPPH_] × 100.

where, A_DPPH_ is the absorbance of the DPPH solution (control) and A_RVE_ is the absorbance of the RVE solution. The concentration at which RVE resulted 50% RSA was termed as IC_50_ value and was calculated using a graph placing % RSA against different RVE concentrations used.

### Experimental animals and experimental design

Male Swiss Albino mice of 42 days old (30–32 g body weight) were acclimatized for 1 week before starting the experiment in a room (temperature of about 25 ± 2 °C and ~ 50% humidity, 12 h dark/light cycle). Drinking water and food were provided *ad libitum*.

Mice were randomly separated into eight groups (*n* = 6). The first (control) group was treated with 0.2 mL of 0.9% NaCl. The next four groups were treated with cisplatin at 2.5 mg/kg for 5 days at an interval of 24 h. After cisplatin administration, one group (second group) was left without any further treatment and assigned as the stressed control group. The third, fourth, and fifth groups were further treated with RVE at 25, 50, and 100 mg/kg, respectively for 5 days. Further three groups were treated with RVE only at 25, 50, and 100 mg/kg, respectively for 5 days. Cisplatin and RVE were dissolved in distilled water. All treatments were given intraperitoneally. After 24 h of last treatment, the animals were euthanized following cervical dislocation [25]. Then the peritoneum was opened with scissor, blood was collected following heart puncture, and kidneys were collected using forceps. Blood was subjected to check the level of creatinine in serum. The kidneys were subjected to evaluating malondialdehyde level and gene expression.

### Measurement of serum creatinie

Serum creatinine was measured using Creatinine Colorimetric Assay Kit-700,460 (Cayman Chemical, USA) following the manufacturer’s protocol provided with the kit.

### Measurement of renal lipid peroxidation

Malondialdehyde (MDA) is an end product of lipid peroxidation in kidney tissue and usually being measured as an indicator of ROS production. However, MDA level was measured in renal tissue according to a prior study [27]. At first, the renal tissue was homogenized in sodium phosphate buffer (0.1 M, pH 7.4). A reaction solution comprising 0.8% thiobarbituric acid (1.5 mL), 8.1% SDS (200 μL), 20% (pH 3.5) acetic acid (1.5 mL), and dH_2_O (600 μL) was added to 100 μL of homogenized tissue, and the mixture was then incubated at 95 °C for 1 h. After cooling, the mixtures were centrifuged at 10,000 g for 10 min at 4 °C and the absorbance of the supernatant was measured at 532 nm with standard 1, 1, 3, 3-tetramethoxypropane. The amount of total protein was measured using the Bradford Protein Assay kit (BIO RAD, USA), and by comparing it with standard *bovine serum albumin* (BSA). The intensity of lipid peroxides was articulated as nanomoles (nM) of MDA per milligram (mg) of protein.

### Real-time polymerase chain reaction (real-time PCR)

Real-time PCR was performed as describes previously [28, 29]. Total RNA from kidney tissue was isolated using TRIzol® reagent (Invitrogen) according to the protocol supplied by the manufacturer. The isolated RNA (1 μg) was then converted into cDNA. Firstly, 2 μL random hexamer (10 μM), 2 μL dNTPs (10 mM), 1 μg RNA, and nuclease-free H O up to 15 μL were taken and incubated for 5 min at 70 °C. The mixture was instantly placed on ice for 2 min. Then 4 μL of 1st strand buffer (5x) and 1 μL M-MLV reverse transcriptase were added in each tube and incubated for 10 min and 50 min at 25 °C and 42 °C, respectively. Finally, the M-MLV reverse-transcriptase enzyme was inactivated by incubating the mixture at 95 °C for 5 min. The synthesized cDNA products were subjected to real-time PCR for quantification of NQO1, p53, and Bcl-2 gene expression using specific primers (Table 1 ). Each reaction (10 μL) was performed in triplicate comprising of 5 μL GoTaq® qPCR Master Mix (2x) (Promega, USA), 0.5 μL (10 mM) of each primer, 3 μL nuclease-free water, and 1 μL cDNA in 48-well reaction plates. Thermal cycling was performed using a real-time PCR machine (Eco™ Real-Time PCR System, Illumine®, USA) with the following cycling conditions: 95 °C for 10 min, followed by 40 cycles of 95 °C for 30 s, 50 °C for 30 s, and 72 °C for 25 s. The specificity of PCR reactions was confirmed by analyzing the melt curve at 95 °C for 15 s, 45 °C for 15 s, and 95 °C for 15 s. The specificity of PCR reactions was confirmed by analyzing the melt curve at 95 °C for 15 s, 45 °C for 15 s, and 95 °C for 15 s. The relative quantification of gene expression was performed using endogenous GAPDH gene as control based on ∆∆Cq method.

**Table 1:** List of primers used in real-time PCR

Gene	Primer	Sequence
NQO1	Forward	5′-TTCTGTGGCTTCCAGGTCTT-3′
	Reverse	5′-AGGCTGCTTGGAGCAAAATA-3′
p53	Forward	5′-GCGTCTTAGAGACAGTTGCCT-3′
	Reverse	5′-GGATAGGTCGGCGGTTCATGC-3′
Bcl-2	Forward	5′-GTGGAGGAGCTCTTCAGGGA-3′
	Reverse	5′-AGGCACCCAGGGTGATGCAA-3′
GAPDH	Forward	5′-GTGGAAGGACTCATGACCACAG-3′
	Reverse	5′-CTGGTGCTCAGTGTAGCCCAG-3′

### Cell culture and treatment

Human renal proximal tubule epithelial cell line, HK-2 cells, were maintained in DMEM supplemented with 10% FBS and antibiotics (50 U/mL of penicillin and 50 μg/mL streptomycin) in an incubator with 5% CO_2_ and 95% humidity at 37 °C.

### H_2_O_2_ measurement assay

H_2_O_2_ level in HK-2 cells was estimated by using ROS-Glo™ H_2_O_2_ Assay kit (Promega, USA) according to the protocol provided by the kit manufacturer. HK-2 cells (1000 cells) in 70 μL DMEM were placed in wells of the 96-well microtiter plate. After allowing attachment of the cells on the well surface, 10 μL DMEM from wells of microtiter plate was replaced with 10 μL cisplatin (25 μM in DMEM) and kept in incubator for 12 h. Then, 10 μL RVE was added to make final concentrations 125, 250, and 500 μg/mL in DMEM and incubated for further 12 h. After that, 20 μL H_2_O_2_ Substrate Solution and 100 μL ROS-Glo™ Detection Solution were added to each well. The reaction was incubated at room temperature for 20 min. Finally, luminescence was measured by using GloMax Luminometer (Promega, USA).

### GC-MS analysis

GC-MS analysis of RVE (dissolved in methanol) was performed as described previously [30] using GCMS-QP2020 (SHIMADZU) comprising an auto-sampler (AOC-20s), an auto-injector (AOC-20i), and a Gas Chromatograph (GC-2010 Plus) interfaced to a Mass Spectrometer equipped with an SH-Rxi-5Sil MS capillary column (30 m × 0.25 μm ID × 0.25 μm DF). The carrier gas Helium was kept at a constant flow rate of 1.72 mL/min, and an injection volume of 5 μL was subjected with 10:1 split ratio. Temperature of the injector was maintained at 220 °C, the ion-source temperature was 280 °C, the oven temperature was programmed from 80 °C (hold for 2 min), with an increase of 5 °C/min to 150 °C (hold time 5.0 min), then 5 °C/min to 280 °C, ending with an 8 min isothermal at 280 °C. Mass spectra were taken at 1.5 kV with a scan interval of 0.5 s and the sample was run at a range of 45–350 m/z. The solvent delay was from 0 to 3 min, and the total GC-MS running time was 55 min. The relative concentration of the detected compounds was measured by comparing its average peak area to the total area. Interpretation of mass-spectrum in GC-MS was performed using the National Institute Standard and Technology (NIST) databases including NIST08, NIST08s, and NIST14.

### Statistical analysis

The statistical analyses were performed by ANOVA following Dunnett’s *T3* test using IBM SPPS (version 20) software. Data are articulated as means ± standard deviation (SD). Significant comparison was considered at *p* < 0.05. All of the graphs were prepared using Microsoft Excel (version 2010).

## Results

### In-vitro antioxidant activity test

**Fig. 1:**
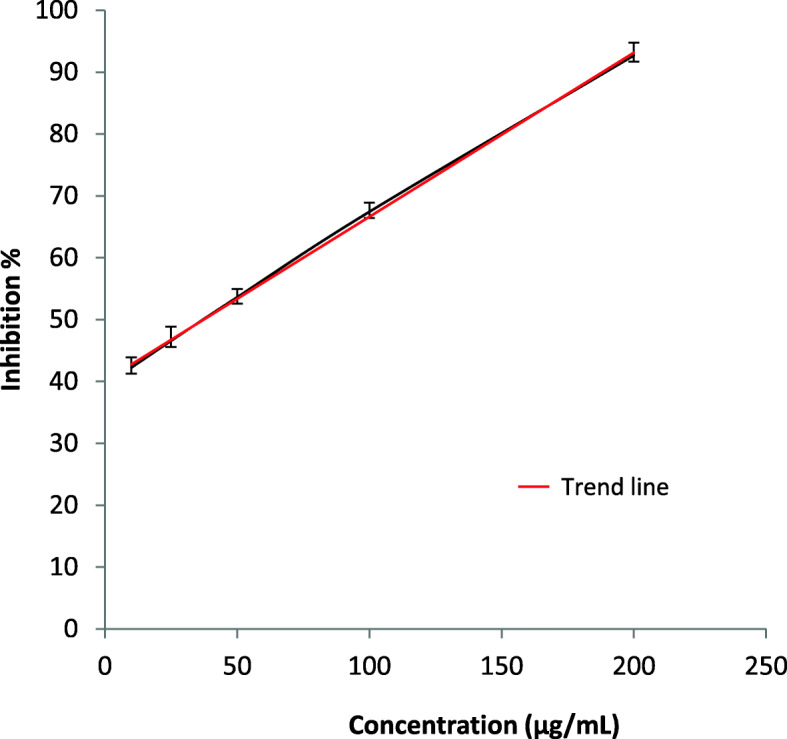
DPPH radical scavenging activity of RVE. RVE scavenged DPPH radical in a dose-dependent manner. Results are mean ± SD (*n* = 3). The mean IC_50_ value of RVE was calculated using regression equation, y = 0.265x + 40.09. The calculated mean IC_50_ value is 37.39 μg/mL (mean ± SD = 37.39 ± 1.89 μg/mL)

Though previously RVE was reported for having antioxidant activity, we rechecked the antioxidant activity of our extract using DPPH free radical scavenging capacity. RVE neutralized DPPH dose-dependently (Fig. 1). RVE revealed considerable in vitro antioxidant activity and the calculated IC_50_ value of RVE was 37.39 ± 1.89 μg/mL.

### Measurement of serum creatinine

Serum creatinine level in mouse was significantly (*p* < 0.05) increased after cisplatin administration (Table 2). Treatment with RVE remarkably (*p* < 0.05) ameliorated creatinine level at in a does-dependedt fashion (Table 2).

**Table 2:** Effects of RVE on serum creatinine level in mouse

Groups	Creatinine (mg/dL) (mean ± SD)
Control	0.54 ± 0.09
Cisplatin (2.5 mg/kg)	2.67 ± 0.21 ^a^
Cisplatin (2.5 mg/kg) + RVE (25 mg/kg)	1.97 ± 0.16 ^b^
Cisplatin (2.5 mg/kg) + RVE (50 mg/kg)	1.38 ± 0.11 ^b^
Cisplatin (2.5 mg/kg) + RVE (100 mg/kg)	0.99 ± 0.07 ^b^

*n* = 6

^a^ significant difference (*p* < 0.05) in respect to control

^b^ significant difference (*p* < 0.05) in respect to cisplatin (2.5 mg/kg) treated group

### Measurement of renal lipid peroxidation

Compared to control, cisplatin considerably (*p* < 0.05) augmented MDA content in renal tissue of mice (Table 3). In contrast, RVE treatment considerably (*p* < 0.05) restored MDA to almost normal in a dose-dependent fashion (Table 3).

**Table 3:** Effects of RVE on MDA levels in mouse kidney tissue

Groups	MDA (nmol/mg protein) (mean ± SD)
Control	2.13 ± 0.72
Cisplatin (2.5 mg/kg)	4.96 ± 1.13 ^a^
Cisplatin (2.5 mg/kg) + RVE (25 mg/kg)	3.05 ± 0.87 ^b^
Cisplatin (2.5 mg/kg) + RVE (50 mg/kg)	2.78 ± 1.03 ^b^
Cisplatin (2.5 mg/kg) + RVE (100 mg/kg)	2.29 ± 1.03 ^b^

*n* = 6

^a^ significant difference (*p* < 0.05) in respect to control

^b^ significant difference (*p* < 0.05) in respect to cisplatin (2.5 mg/kg) treated group

### Real-time PCR

**Fig. 2:**
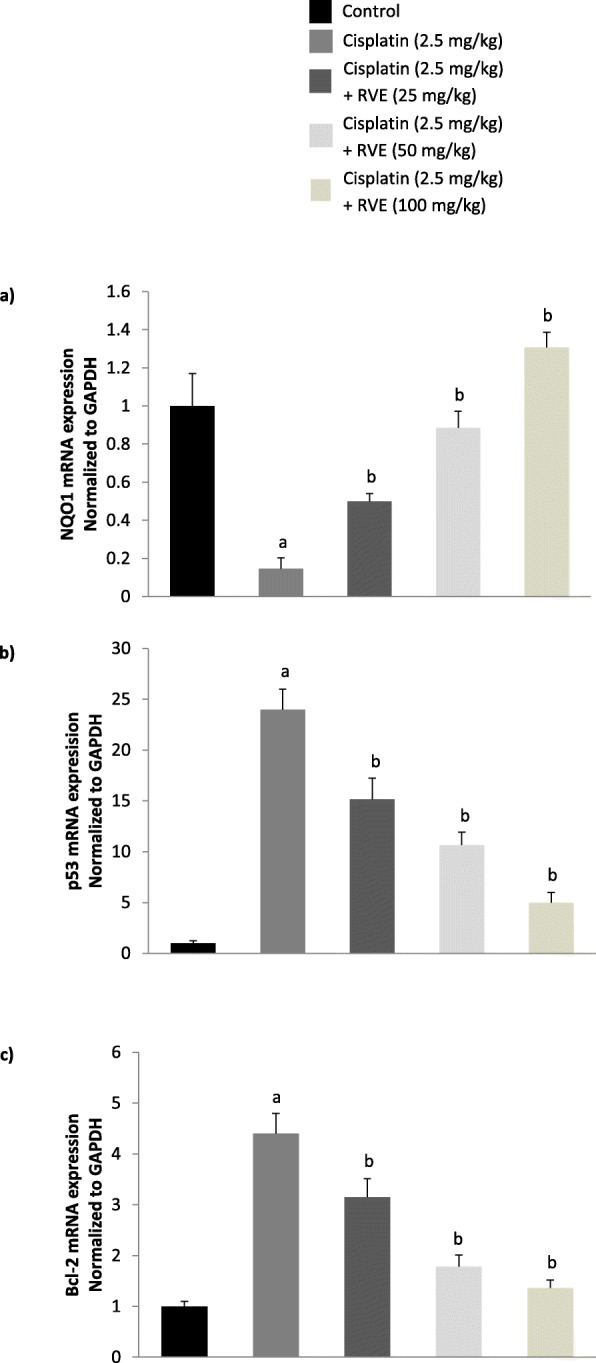
Ameliorative effect of RVE on expression of NQO1, p53, and Bcl-2 mRNA in mice kidney tissue. a) mRNA quantity of NQO1 b) mRNA quantity of p53 c) mRNA quantity of Bcl-2. Results are mean ± SD (*n* = 6). ^a^ significant difference (*p* < 0.05) in respect to control. ^b^ significant difference (*p* < 0.05) in respect to cisplatin (2.5 mg/kg) treated group

**Fig. 3:**
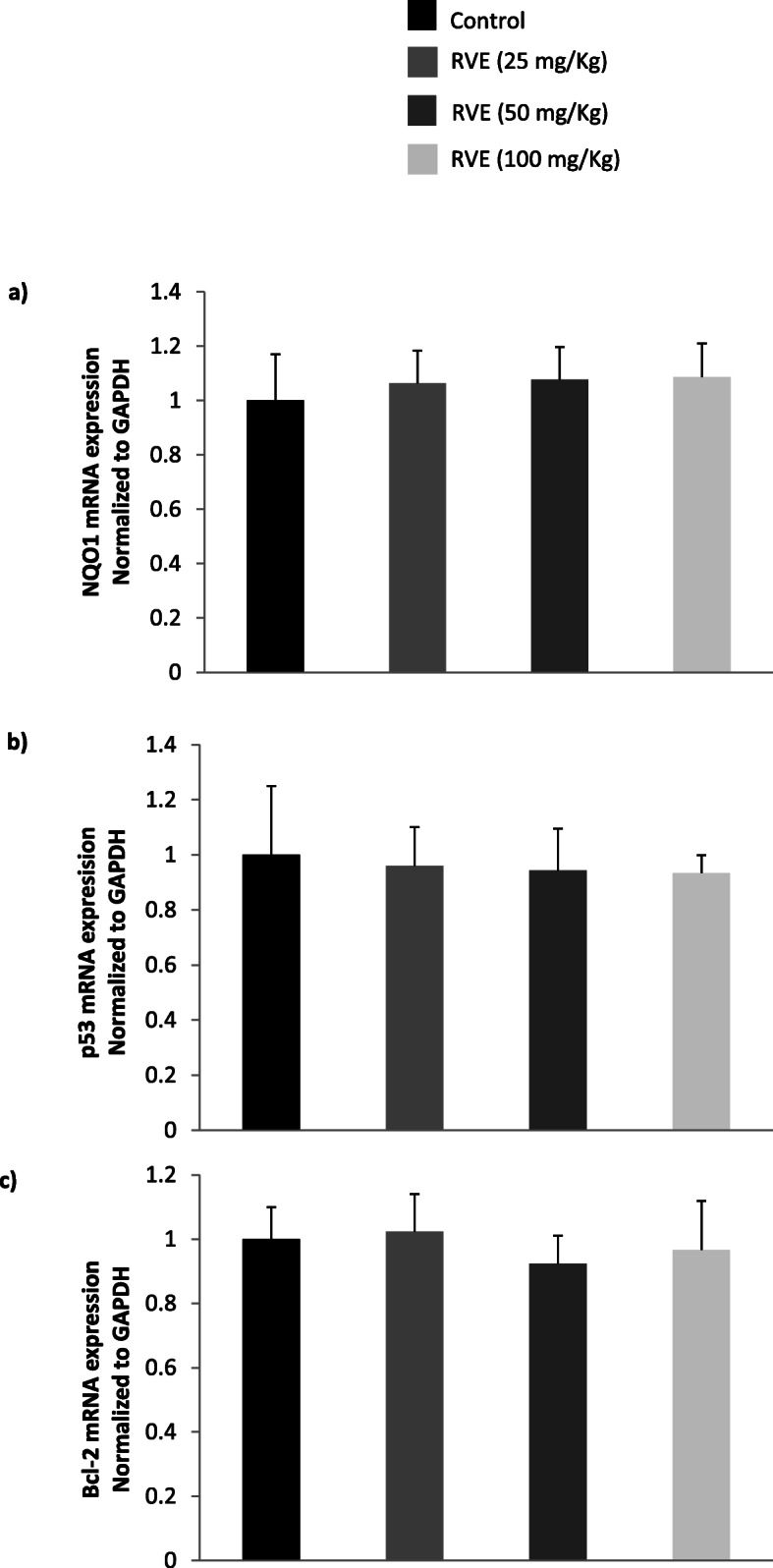
Effect of RVE alone on the expression of NQO1, p53, and Bcl-2 mRNA in mice kidney tissue. a) mRNA quantity of NQO1 b) mRNA quantity of p53 c) mRNA quantity of Bcl-2. Results are mean ± SD (*n* = 6). No significant difference (*p* > 0.05) was found in only RVE treated group compared to the control

Cisplatin significantly (*p* < 0.05) decreased NQO1 mRNA expression by 0.15-fold and increased p53 and Bcl-2 mRNA expression by 24 and 4.2-fold, respectively (Fig. 2). RVE considerably (*p* < 0.05) mitigated NQO1, p53, and Bcl-2 mRNA expression in a dose-dependent fashion (Fig. 2). Compared to only cisplatin-treated group, NQO1 mRNA expression was increased by 3.57, 6.36, and 9.28-fold at 25, 50, and 100 mg/kg RVE, respectively (Fig. 2a). Again, p53 mRNA expression was decreased by 0.63, 0.46, and 0.21-fold at 25, 50, and 100 mg/kg RVE, respectively (Fig. 2b). Bcl-2 mRNA expression was also reduced by 0.71, 0.40, and 0.32-fold at 25, 50, and 100 mg/kg RVE, respectively (Fig. 2c). But, no significant (*p* > 0.05) changes were found in the expression level of NQO1, p53, and Bcl-2 genes due to treatment with RVE (Fig. 3).

### H_2_O_2_ measurement assay

**Fig. 4:**
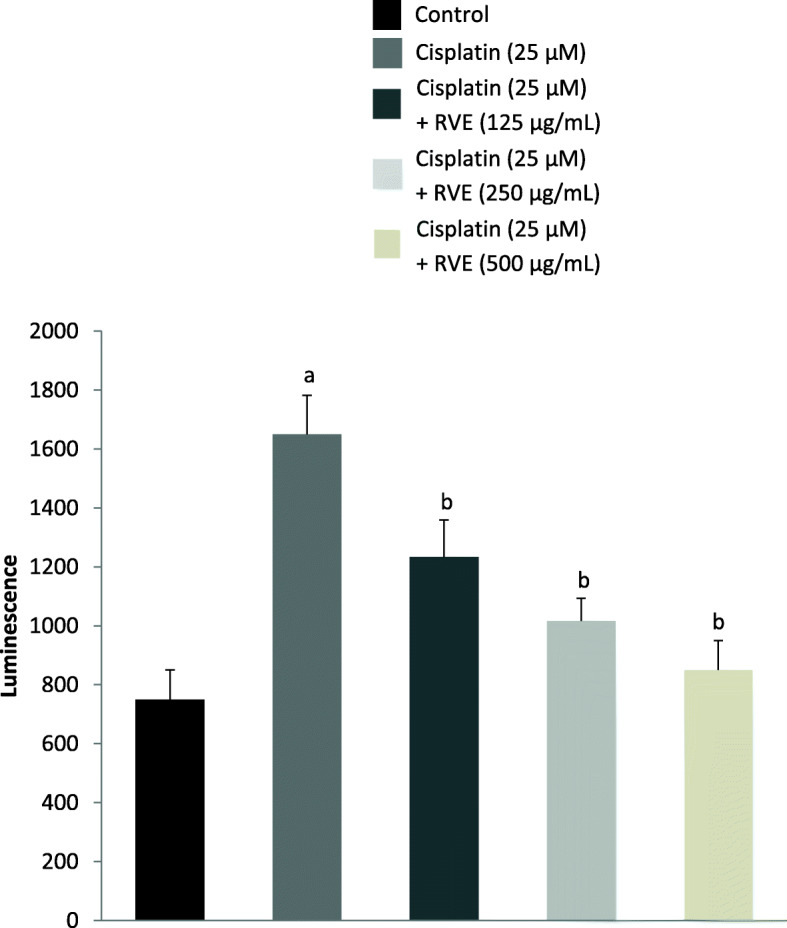
Effect of RVE on H_2_O_2_ level in HK-2 cells. H_2_O_2_ level is proportionate to luminescence. Results are mean ± SD (*n* = 6). ^a^ significant difference (*p* < 0.05) in respect to control. ^b^ significant difference (*p* < 0.05) in respect to cisplatin (25 μM) treated group

In the H_2_O_2_ measurement assay, the H_2_O_2_ level was considered proportionate to luminescence. Administration of cisplatin significantly (*p* < 0.05) increased H_2_O_2_ level by 2.2-fold (Fig. 4). Treatment with RVE considerably decreased (*p* < 0.05) H_2_O_2_ level by 0.25, 0.38, and 0.49-fold at 125, 250, and 500 μg/mL, correspondingly.

### GC-MS analysis

**Fig. 5:**
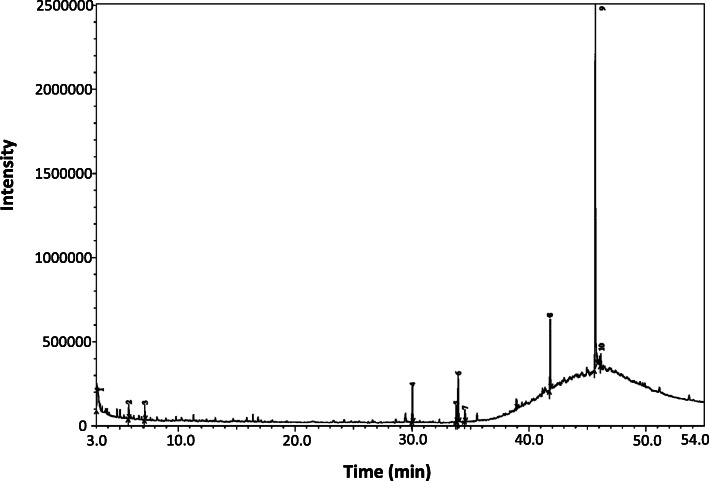
GC-MS chromatogram of RVE

A total of 10 compounds (Table 4 and Fig. 5) including “Isoborneol, pentamethyldisilanyl ether (sesquiterpene alcohol)”, “Thymine (pyrimidine nucleobase)”, “4H-Pyran-4-one, 2,3-dihydro-3,5-dihydroxy-6-methyl- (saponin)”, “Hexadecanoic acid, methyl ester (fatty acid methyl ester)”, “9,12-Octadecadienoic acid, methyl ester (fatty acid methyl ester)”, “9-Octadecenoic acid (Z)-, methyl ester (fatty acid methyl ester)”, “Methyl stearate (fatty acid methyl ester)”, “Diisooctyl phthalate (ester)”, “13-Docosenamide, (Z)- (alkaloid)”, and “Squalene (triterpene)” were detected in RVE.

**Table 4:** List of compounds in RVE identified by using GC-MS

Peak no.	Compounds	Ret. time	Molecular formula	Molecular weight	Con. %
1	Isoborneol, pentamethyldisilanyl ether	3.018	C_15_H_32_OSi_2_	284.59	0.62
2	Thymine	5.769	C_5_H_6_N_2_O_2_	126.115	4.32
3	4H-Pyran-4-one, 2,3-dihydro-3,5-dihydroxy-6-methyl-	7.144	C_6_H_8_O_4_	144.126	2.08
4	Hexadecanoic acid, methyl ester	30.020	C_17_H_34_O_2_	270.457	8.91
5	9,12-Octadecadienoic acid, methyl ester	33.785	C_19_H_34_O_2_	294.479	1.58
6	9-Octadecenoic acid (Z)-, methyl ester	33.942	C_19_H_36_O_2_	296.495	5.77
7	Methyl stearate	34.519	C_13_H_26_O_2_	214.349	2.65
8	Diisooctyl phthalate	41.810	C_24_H_38_O_4_	390.564	18.62
9	13-Docosenamide, (Z)-	45.678	C_22_H_43_O_4_	337.592	51.99
10	Squalene	46.146	C_30_H_50_	410.73	3.45

## Discussion

Natural and synthetic antioxidants have been comprehensively studied and revealed to be functional for either the prevention or amelioration of toxicity in animal physiology [31]. Antioxidant supplements are the component developed either by chemical synthesis or by extraction from natural foods but these are not identical in composition as antioxidants available in food [5]. Therefore, the opinions are separated over time whether or not synthetic antioxidants give similar health benefits as natural antioxidants [32]. The urge is coming up to decrease the use of synthetic antioxidant supplements and seeking alternative, cheap, renewable, natural, and possibly safer sources of effective natural antioxidants.

One of the most important mechanisms is the nuclear factor erythroid-2 related factor-2 (Nrf2) pathway that generally protects cells from oxidative stress-induced by exogenous or endogenous stressors [27]. The effective antioxidants induce expression of Nrf2, which further moves into the nucleus and binds to antioxidant response element (ARE) that provokes expression of phase II detoxifying and antioxidant gene NQO1 [33, 34]. NQO1 is widely and differentially expressed in a tissue-specific manner. NQO1 is a cytosolic antioxidant flavoprotein that catalyzes the 2-electron reduction of quinones to hydroquinones, resulting in detoxification of the electrophiles and anticipation of redox cycling [35]. According to a previous study [36], β-lapachone activates NQO1, which further increases intracellular NAD^+^ level and protects the kidney from cisplatin-induced acute injury.

Cisplatin is known to induced damage in the glomerular filtration membrane through oxidative stress, inflammation, and apoptosis which altogether lead to reduce glomerular filtration rate and loss of normal membrane permeability [37]. Therefore, the serum creatinine level was increased. Serum creatinine is one of the potential renal functionality markers. Cisplatin treatment increased MDA content in kidney tissue which is a secondary product of lipid peroxidation and this report is constant with previous studies [27, 35, 37, 38]. Treatment with RVE significantly reduced MDA content in kidney tissue. At the same time, the creatinine level was also decreased indicating the ameliorative effect of RVE.

Besides, NQO1 expression was significantly decreased, and p53 and Bcl-2 expression were significantly increased after exposure to cisplatin. In terms of NQO1 and p53 expression in vivo, our result is consistent with a prior study [13]. A recent study [39] showed that cisplatin significantly decreased expression of Bcl-2 in the kidney of mouse, but surprisingly we found elevated expression. This difference may be the result of dose difference [40] as Mohamed and colleagues [39] used 8 mg/kg for 12 days, whereas we used 2.5 mg/kg for only 5 days. Another study [41] stated that cisplatin may increase the expression of Bcl-2 at a dose when it is non-cytotoxic. Again, increased Bcl-2 expression sensitizes cells towards oxidative stress [40].

However, expression of p53 is low at normal physiological conditions, but expected to be up-regulated once treated with cisplatin because this platinum-based chemotherapeutic agent activates p53 dependent apoptotic pathway. Once we treated mice with cisplatin, p53 was significantly increased in mice kidneys compared to control. Another proto-oncogene Bcl-2 is also correlated with NQO1 expression level. Mimicking the p53 expression, we also found that the Bcl-2 level was increased with cisplatin treatment, but recovered significantly upon treatment with RVE. This is possibly, in response to oxidative stress, the p53 gene gets activated and results in arresting cell cycle, senescence, or apoptosis [6]. With detoxification, NQO1 overexpression is often considered to be correlated with apoptosis in cancer cell [10], though the underlying mechanism of apoptosis and the overexpression of NQO1 is still controversial. Moreover, in hepatocellular carcinoma, NQO1 overexpression decreases the Bcl-2 expression [10]. Proto-oncogene Bcl-2 also has p53 like correlation with NQO1 expression. p53 is a sequence-specific transcription factor that gets activated by numerous types of cellular stress [42], whereas Bcl-2 overexpression acted as mitochondrial pore-stabilizer to facilitate cytochrome-C release upon oxidative stress [12]. In our case, we checked both of the gene responses with cisplatin treatment in normal kidney tissue and found their increased expression. RVE treatment reverted the expression of p53 and Bcl-2 to almost normal in a dose-dependent fashion. This type of Bcl-2 expression abrogation was also shown using ROS scavenger Trolox [43]. Moreover, H_2_O_2_ level was also markedly increased in HK-2 cells due to treatment with cisplatin in vitro. After treatment with RVE, the H_2_O_2_ level was significantly restored to around normal. This is maybe due to the effect of RVE treatment that increased NQO1 expression [44], which exerts protection against oxidative stress [6].

The GC-MS chromatogram confirmed the existence of ten compounds in RVE. Among these, “4H-Pyran-4-one, 2, 3-dihydro-3,5-dihydroxy-6-methyl-”, “Hexadecanoic acid” and “Squalene” are well-known antioxidants[45–47]. These three compounds altogether possibly exerted a synergistic nephroprotective effect. Previous studies also reported about induction of NQO1 expression by vitamin A [48], vitamin C [49], vitamin E [50], flavonoids [51], and polyphenols [50]. Therefore, this report suggests elucidating whether this particular extract contains anything among vitamin A, vitamin C, vitamin E, flavonoids, and polyphenol, or not.

## Conclusion

The overall finding suggests that RVE is physiologically effective in protecting kidneys from cisplatin-induced damage. Therefore, it is crucial to elucidate the exact compounds responsible for mitigating cisplatin-induced nephrotoxicity which may become beneficial for human application.

## Data Availability

All relevant data are available and could be provided upon request to the corresponding author.
